# Measurement of bony anatomical parameters of the distal ulna based on healthy adult data: A cross-sectional study

**DOI:** 10.3389/fsurg.2023.1120030

**Published:** 2023-03-15

**Authors:** Dengchen Li, Xiaolin Li, Zexin Hou, Fengman Pan

**Affiliations:** ^1^ School of Medicine, Yangtze University, Jingzhou, China; ^2^ School of Medicine, Yangtze University, Jingzhou, China; ^3^ College of Acupuncture and Orthopedics, Hubei University of Chinese Medicine, Wuhan China; ^4^ Department of Medical Imaging, The First Affiliated Hospital of Yangtze University, Jingzhou, China; ^5^ Department of Medical Imaging, First People's Hospital of Jingzhou, Jingzhou, China

**Keywords:** distal ulna, digital anatomy, mimics software, wrist, adult

## Abstract

**Purpose:**

This study sought to conduct several three-dimensional measurements of the distal ulna in healthy Han Chinese, providing the anatomical basis for the diagnosis and treatment of hand trauma, distal ulnar disorders, and the design of wrist prostheses.

**Methods:**

50 Han Chinese men and women that underwent computed tomography (CT) scans of the distal ulnar carpus were included in the present study. A three-dimensional digital model of the distal ulna was reconstructed using Mimics software. Moreover, the anatomical data of 10 indicators were measured using MIMICS software. Each index data was measured by 2 investigators independently, and the average value was taken. The data were stratified and compared between left and right sides and men and women.

**Results:**

A 3D digital model of the distal ulnar bone with a realistic shape was reconstructed. The 10 anatomical parameters measured are as follows: The length of the ulnar styloid process (posterior anterior), The length of the ulnar styloid process(anterior and posterior); the transverse diameter of the ulnar head; the anteroposterior diameter of the ulnar head. The radial inclination angle of the ulna; the ulnar inclination angle; the distal space between the ulna and radius; the ulnar notch angle of the lower radius. The anterior and posterior diameters of the ulnar notch of the lower radius, and the superior and inferior diameters of the ulnar notch of the lower radius. Statistical analysis showed no significant difference after stratification by laterality and gender.

**Conclusion:**

our findings can providing the anatomical basis for the diagnosis and treatment of hand trauma, distal ulnar disorders and further improve currently available wrist joint prostheses.

**Type of Study:**

Observational, Cross-sectional study, LOE: Level II

## 1. Introduction

The wrist joint is a complex and delicate joint composed of the distal ulnar radius, eight carpal bones and the proximal end of the metacarpal bone, which is used most frequently in daily life and work and is, therefore, prone to injury. Although the radius dominates the proximal end, the ulna also plays an important role in the movement of the entire wrist joint. It has long been controversial whether to operate on the distal ulna in patients with distal radius fractures combined with distal ulnar fractures, but an increasing body of evidence from domestic and western studies suggests that operating on distal ulna fractures combined with distal radius fractures can reduce the incidence of complications, promote fracture healing, and significantly improve the wrist joint function (1–6). This finding proves that the distal ulna is a very important part of the wrist joint for normal functional activity, and the importance of the distal ulna in the wrist joint is increasingly appreciated. In severe wrist injuries, late wrist replacement is an effective way to save the function of the patient's hand, but the wrist joint prosthesis is not as good as it should be, resulting in a poor outcome of wrist joint replacement. The diagnosis and treatment of wrist disorders and the design of wrist prostheses require detailed knowledge of the anatomy of the bones that make up the wrist joint. In this respect, Moore Douglas C et al. have started to establish a database of carpal anatomy and wrist motion (7). Mona Hassan Mohammed Ali et al. (8) used 300 normal radiographs for posterior-anterior anatomical data of the wrist joint to establish a database of normal carpal anatomy in Egyptians. A domestic team represented by Professor Xu Yongqing used cadaveric specimens to measure and analyze the bones in wrist joints (9–12), yielding bony anatomical data of the carpal bones such as the lunar bone, the hook bone, the cephalic bone, and the large and small polygonal bones. Ryan Lohre (13) used x-rays to photograph the wrist joint in 60 patients and measured the distal ulnar Diaphyseal angle, but the x-rays were less accurate than CT and the study's measurements were not comprehensive enough. Due to the potential influence of different ethnic groups, data from western studies cannot be applied to the Chinese population. To our knowledge, no studies have hitherto been reported on the distal ulna abroad and in China.

The role of the distal ulna in the overall wrist joint has received increasing attention over the years, while the fine anatomy of the distal ulna has been understudied. Basic research is urgently needed to further improve the diagnosis and treatment of distal ulnar disorders and to design safer and more effective wrist prostheses. A comprehensive study of the distal ulna anatomy in normal Han Chinese is an important part of the relevant basic research. In this paper, we applied CT scan data of the wrist joint, including the distal ulna, in a normal Han Chinese population to reconstruct a 3D digital model of the wrist joint including the distal ulna, to provide a more comprehensive set of anatomical data of the distal ulna by performing 3D stereoscopic measurements of the structures related to the distal ulna.

## 2. Data and methods

### 2.1. Object of the study

Based on previous literature research, Combined with statistical calculation, The specimens for our study was 100, Fifty Han Chinese normal males and 50 females each,selected by random sampling (provided informed consent and approved by the ethics committee of the university), with a mean age of 37.5 (20–50 years), mean height of 168.4 (145–190 cm) and weight of 42–80 kg, with no deformity and fracture of the bilateral wrist joints, were included in this study. Anterior, oblique and lateral radiographs of bilateral wrist joints were obtained to confirm that no bone abnormalities were found in the selected volunteers.

### 2.2. Software and hardware equipment

64-row slice spiral CT (GE Healthcare, USA), MIMICS 10.0 software (Materialise, Belgium).

### 2.3. CT raw data acquisition

CT scans were performed in the Department of Radiology of the First People's Hospital affiliated with our hospital, and fine-cut uninterrupted CT scans were performed on each volunteer's bilateral wrist joints, with a scanning range of 10.0 cm above the distal radial ulnar joint to the end of the fingers, The scan parameters included: selection of bone tissue window, voltage 120 kV, 65 Ma, pixel size 0.43 mm, layer thickness 0.625 mm, matrix 512 × 512, and the scanned images were saved in DICOM format on disk.

### 2.4. 3D Modeling of the distal ulnar radius and hand

The CT data of patients were input into the MIMICS software. After determining the orientation, the bone threshold was defined between 226-1950HU. The distal ulnar and radius were selected as the modeling range and underwent regional enhancement. Noise reduction was conducted to remove redundant data, and 3D calculations were conducted to generate the 3D model of the distal ulnar radius and hand.

### 2.5. Measurement of bony anatomical parameters of the distal ulna

#### 2.5.1. Measurement indexes

Based on previous literature research, combined with our clinical experience, 10 bony anatomical datas of distal ulna were measured. (1) ulnar styloid length (posterior-anterior position) (2) ulnar styloid length (anterior-posterior position) (3) transverse ulnar head diameter (4) anterior-posterior ulnar head diameter (5) ulnar radial tilt angle (6) ulnar ulnar tilt angle (7) distal ulnar radial gap (8) ulnar notch angle of the lower radius (9) anterior-posterior radial notch diameter (10) upper and lower ulnar notch diameter of the lower radius. More details on the positioning are shown in Table 1.

**Table 1:** Distal ulna measurement index and positioning criteria.^a^

Measurement indicators	Positioning criteria
Length of ulnar styloid process (posterior-anterior position)	The parallel distance from the ulnar carpal surface to the tip of the ulnar styloid process
Length of ulnar styloid process (anterior-posterior position)	The parallel distance from the ulnar carpal surface to the tip of the ulnar styloid process
Transverse diameter of ulnar head	The maximum radial to ulnar diameter of the ulnar head at the lower end of the ulna
Anteroposterior diameter of the ulnar head	Maximum diameter of the dorsal to palmar aspect of the ulnar head at the lower end of the ulna
Radial tilt angle of ulna	Orthotropic display of the lower ulnar segment measuring the radial to ulnar angle of inclination of the lower ulnar segment
Ulnar tilt angle	Orthotropic display of the inferior ulnar segment measuring the ulnar to radial angle of inclination of the inferior ulnar segment
Distal space of ulna and radius	The distance from the ulnar side of the radial joint to the radial side of the ulnar joint.
Angle of ulnar notch at the lower end of radius	Transverse angle of ulnar notch at the lower end of radius
Anteroposterior diameter of ulnar notch at the lower end of radius	Distance from palmar margin to dorsal margin of ulnar notch at the lower end of radius
Superior and inferior diameter of ulnar notch at the lower end of radius	The distance from the upper edge to the lower edge of the ulnar notch at the lower end of the radius

^a^ Distal ulna measurement index and positioning criteria.

#### 2.5.2. Measurement method

**Figure 1:**
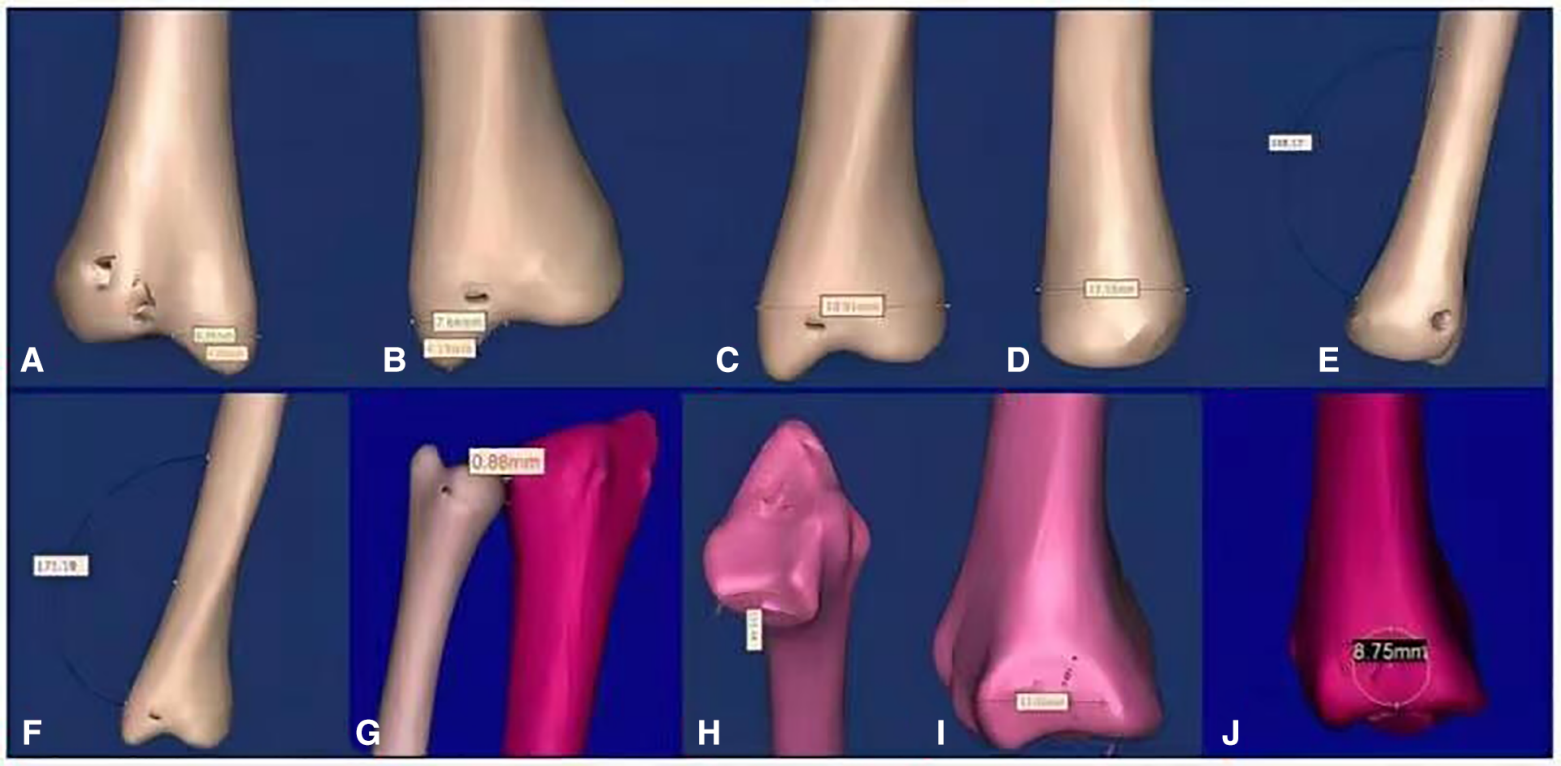
Measurement chart ((**A**) ulnar styloid length (posterior-anterior position), (**B**) ulnar styloid length (anterior-posterior position), (**C**) transverse ulnar head diameter, (**D**) anterior-posterior ulnar head diameter, (**E**) ulnar radial inclination angle, (**F**) ulnar ulnar inclination angle, (**G**) distal ulnar radial clearance, (**H**) inferior radial ulnar notch angle, (**I**) inferior radial ulnar notch anterior-posterior diameter, (**J**) inferior radial ulnar notch superior-inferior diameter).

MIMICS software was used to measure the distance or angle using the measure 3D distance or measure 3D angle tools, respectively. The measurement method is shown in Figure 1. To minimize the measurement errors caused by human factors, Each index data was measured by 2 people independently, the computer automatically generated the measurements.the average value was taken, and the results were approximated to an accuracy of 0.01. The measurement fixing points and methods are shown in Figure 1.

### 2.6. Statistical treatment

Statistical Package for Social Sciences (SPSS 20.0 statistical package) was used for analysis, and data were expressed as mean ± standard deviation. Data from each group were stratified according to laterality (left vs. right side) and gender (men vs. women) and compared using an independent sample t-test. A *P*-value <0.05 was statistically significant.

## 3. Results

**Figure 2:**
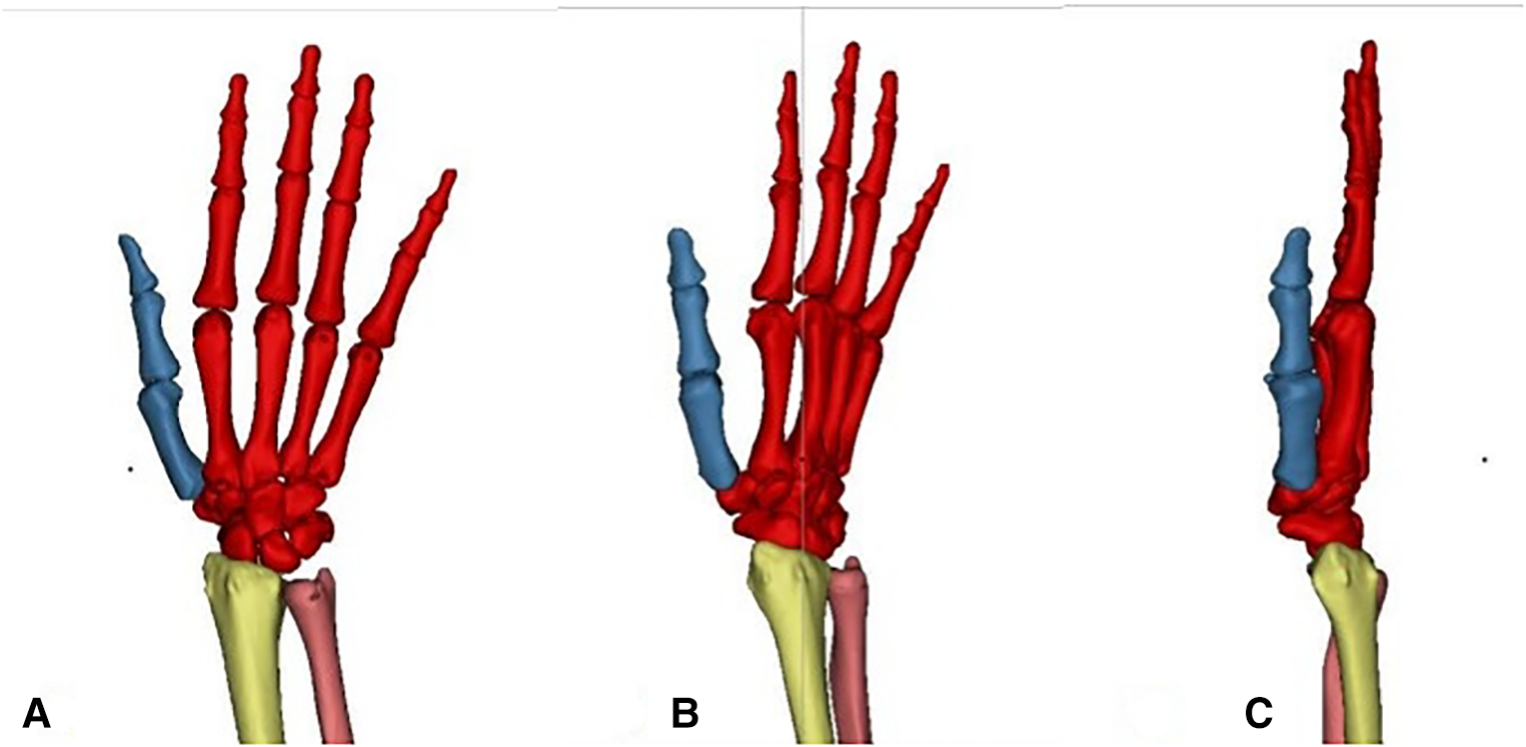
3d view of the whole wrist joint [(**A**) positive phase (**B**) oblique 45° phase (**C**) side phase].

A more comprehensive 3D reconstruction model of the whole wrist joint of a healthy Han Chinese was obtained, including the distal ulnar radius, carpal bones, metacarpals and all phalanges (Figure 2).

More detailed anatomical data of the distal ulna were obtained, as shown in Table 2.

**Table 2:** Left-right comparison of measurement data of the inferior ulna.

Position	Length of ulnar styloid process (posterior-anterior position) mm	Length of ulnar styloid process (anterior-posterior position) mm	Transverse diameter of the ulnar head mm	Anteroposterior diameter of the ulnar head mm	Radial tilt angle of ulna mm	Ulnar tilt angle mm	Distal space of ulna and radius mm	Angle of ulnar notch at the lower end of radius mm	Anteroposterior diameter of ulnar notch at the lower end of radius mm	Superior and inferior diameter of ulnar notch at the lower end of radius mm
Left side (*n* = 100)	4.46 ± 0.58	4.55 ± 0.51	18.82 ± 0.40	16.44 ± 0.98	167.26 ± 2.131	170.92 ± 0.60	0.90 ± 0.35	165.16 ± 5.27	12.93 ± 0.57	7.76 ± 0.17
Right side (*n* = 100)	4.41 ± 0.80	4.66 ± 0.45	18.81 ± 0.40	16.44 ± 0.96	166.82 ± 2.41	170.80 ± 3.04	0.91 ± 0.04	165.83 ± 2.68	12.84 ± 0.42	7.74 ± 0.16
*t*-value	0.46	1.51	0.28	0.03	1.35	0.40	0.25	1.13	1.25	0.88
*p*-value	0.65	0.13	0.78	0.97	0.18	0.69	0.80	0.26	0.21	0.38
	>0.05	>0.05	>0.05	>0.05	>0.05	>0.05	>0.05	>0.05	>0.05	>0.05

Left-right comparison of measurement data of the inferior ulna.

Statistical analysis revealed no significant differences between the left and right sides in ulna-related indexes (Table 2). There was no statistically significant difference between men and women (Table 3).

**Table 3:** Comparison of male and female distal ulna anatomy.

Gender	Length of ulnar styloid process (posterior-anterior position) mm		Length of ulnar styloid process (anterior-posterior position) mm		Transverse diameter of the ulnar head mm		Anteroposterior diameter of the ulnar head mm		Radial tilt angle of ulna mm	
	left	right	left	right	left	right	left	right	left	right
Male (*n *= 50)	4.38 ± 0.62	4.29 ± 0.78	4.63 ± 0.52	4.69 ± 0.41	18.77 ± 0.46	18.78 ± 0.44	16.41 ± 0.92	16.60 ± 0.70	167.31 ± 2.15	166.51 ± 2.82
Female (*n *= 50)	4.53 ± 0.53	4.54 ± 0.82	4.48 ± 0.49	4.62 ± 0.48	18.87 ± 0.34	18.83 ± 0.35	16.47 ± 1.04	16.27 ± 1.14	167.20 ± 2.13	167.14 ± 1.90
*t*-value	1.34	1.57	1.45	0.81	1.25	0.65	0.28	1.74	0.26	1.32
*p*-value	0.18	0.12	0.15	0.42	0.22	0.52	0.78	0.09	0.80	0.19
	>0.05	>0.05	>0.05	>0.05	>0.05	>0.05	>0.05	>0.05	>0.05	>0.05
Gender	Ulnar tilt angle mm		Distal space of ulna and radius mm		Angle of ulnar notch at the lower end of radius mm		Anteroposterior diameter of ulnar notch at the lower end of radius mm		Superior and inferior diameter of ulnar notch at the lower end of radius mm	
	left	right	left	right	left	right	left	right	left	right
Male (*n *= 50)	170.96 ± 0.65	170.37 ± 2.18	0.84 ± 0.27	0.91 ± 0.04	164.50 ± 6.73	165.47 ± 2.56	12.93 ± 0.62	12.83 ± 0.43	7.78 ± 0.16	7.77 ± 0.19
Female (*n *= 50)	170.89 ± 0.56	171.23 ± 3.67	0.96 ± 0.42	0.91 ± 0.04	165.82 ± 3.14	166.19 ± 2.77	12.94 ± 0.53	12.86 ± 0.42	7.74 ± 0.17	7.71 ± 0.12
*t*-value	0.57	1.42	1.72	0.14	1.26	1.36	0.06	0.49	1.21	1.73
*p*-value	0.57	0.16	0.09	0.89	0.21	0.18	0.95	0.66	0.23	0.09
	>0.05	>0.05	>0.05	>0.05	>0.05	>0.05	>0.05	>0.05	>0.05	>0.05

Comparison of male and female distal ulna anatomy.

## 4. Discussion

Current evidence suggests that hand injuries remain highly in common worldwide, especially in China, where industrial automation is not very high. Moreover, the requirements for post-injury function following hand injuries have increased. Although hand surgeons in China have rich clinical experience, the functional recovery of patients with wrist diseases remains unsatisfactory because of the complex characteristics of anatomy, biomechanics and injury of the wrist (14–16). The clinical incidence of distal radius fracture is high, especially in postmenopausal women because of osteoporosis. Indeed, it has been established that in this patient population, distal radius fractures are rife and generally associated with ulnar styloid process fractures. Over the years, the treatment of this patient population has predominantly focused on the distal radius with little emphasis placed on the ulnar styloid process. In recent years, much emphasis has been placed on improving the function of the wrist joint. An increasing body of evidence suggests that treating the ulnar styloid process is very important to restore the function of the wrist joint (17–26). It has been established that the ulnar styloid process is relatively small, irrespective of the measurement approach, with a length of only about 4.5-inche. In such cases, internal fixation is often difficult and mastering the direction of the needle, and the size of the screw is often challenging. Indeed, the recovery of hand function following trauma and the treatment of the ulnar styloid process after distal radius fracture requires detailed anatomical knowledge of the wrist joint and ulna to provide the theoretical basis for clinical practice.

In this study, 10 anatomical parameters of the distal ulna, including the ulnar head, styloid process of ulna, inferior radioulnar joint and distal articular surface of ulna, were observed. Importantly, we found that the end of the ulna is not a straight tube but is tilted towards the radial and ulnar sides. The radial tilt angle of the ulna is about 166°, and the ulnar tilt angle is about 170°. Herein, we investigated the radial and ulnar tilt angles of the ulna, providing a reference for the reduction and fixation of distal ulnar fractures, especially for the pre-bending angle of the internal fixation plate. Moreover, the normal length of the styloid process of the ulna was measured from the anterior and posterior directions, and it was found that the length was the same in both positions, Its length is about 4.5 mm., which indicated that the styloid process of the ulna could be entered from the metacarpal or dorsal side when fixing the styloid process of the ulna. Clinically, distal ulnar variation is common. It is widely acknowledged that ulnar impingement syndrome occurs when there is a positive ulnar variance (the distal ulna is longer than the radius) (27). However, a negative ulnar variance (the ulna is shorter than the radius) can lead to ischemic necrosis of the lunate bone (28). Accordingly, during clinical diagnosis and orthopedic osteotomy, the tilt angle of the distal ulna, the transverse diameter of the ulnar head, and the anterior and posterior diameters should be considered; restoration to its normal anatomic position is the key to successful surgical treatment. Dislocation of the lower ulnar and radial joint is a common wrist injury complication. The distal space of the ulna and radius is an important index for diagnosing dislocation and judging reduction. Due to the lack of cartilage on the x-ray film, there is a certain gap between the distal end of the ulna and the radius during normal photography. According to the measurements of the present study, the gap will not exceed one inch. A dislocation should be strongly suspected if it exceeds one inch and is accompanied by clinical symptoms. Moreover, it can guide clinical treatment and evaluate the curative effect of lower radial and ulnar joint dislocation. The four anatomical variations of the distal sigmoid notch of the radius were correlated with the stability of the inferior radioulnar joint. The instability rate of the distal radioulnar joint is higher in the Flatt type, S type and Ski type sigmoid notch., with the lowest rates associated with the C type (29). In this study, the parameters such as the angle of the ulnar notch at the lower end of the radius, the anterior and posterior diameter of the ulnar notch at the lower end of the radius, and the upper and lower diameter of the ulnar notch at the lower end of the radius were measured, which provided an accurate anatomical basis for judging the anatomical variation and the degree of variation of the sigmoid notch.

Moreover, 10 parameters were measured, no significant difference was found between the right and left sides and males and females, suggesting that laterality and gender are irrelevant during diagnosing and treating diseases related to the distal ulna. For serious wrist diseases such as wrist injury, suppurative arthritis and rheumatoid arthritis, compared with wrist fusion and proximal row carpectomy, wrist replacement brings many advantages, including better pain relief and maximum preservation of wrist function (30). However, it is not widely used in the clinic since it is prone to complications such as joint prosthesis loosening, sinking, dislocation and periprosthetic fracture (31,32). In addition to the complex structure of the wrist, the lack of joint prosthesis design is also an important reason. More anatomical studies are warranted to design a more suitable prosthesis, especially for Chinese patients. The measurement and study of the relevant parameters of the bony structure of the distal ulna, combined with the anatomical data of other bones of the wrist, can provide a bony structural basis for designing a prosthesis that is adequate for Chinese patients.

In the present study, based on the original CT scan data of normal healthy subjects, the digital visual model of the distal ulna of normal adults was established using the MIMICS 3D modeling software; the relevant anatomical datas were measured by the two-dimensional and three-dimensional measurement functions of MIMICS software. During measuring it was only necessary for the researcher to determine the starting and ending points, the computer generated the measurements automatically. The study of anatomical measurement was carried out using digital software based on the original datas of the normal human body. Compared with the traditional autopsy measurement, this method is more reproducible, the data is closer to the truth, and the fineness and credibility are improved. At the same time, a wider data source is available, and a large sample study can be carried out. In this study, a three-dimensional model of the whole wrist joint was established, and on this basis, 10 bone anatomical parameters of the distal ulna were measured, which is more comprehensive in previous studies, and the anatomical data obtained were relatively more detailed. Moreover, the number of selected specimens was small, emphasizing the need for more studies.

## Data Availability

The original contributions presented in the study are included in the article/Supplementary Material, further inquiries can be directed to the corresponding author/s.
